# Alcohol Abuse Promotes Changes in Non-Synaptic Epileptiform Activity with Concomitant Expression Changes in Cotransporters and Glial Cells

**DOI:** 10.1371/journal.pone.0078854

**Published:** 2013-11-13

**Authors:** Luiz Eduardo Canton Santos, Gilcélio Amaral da Silveira, Victor Diego Cupertino Costa, Aline Gisele Batista, Ana Paula Madureira, Antônio Márcio Rodrigues, Carla Alessandra Scorza, Henrique Alves Amorim, Ricardo Mário Arida, Mario Antônio Duarte, Fúlvio Alexandre Scorza, Esper Abrão Cavalheiro, Antônio-Carlos Guimarães de Almeida

**Affiliations:** 1 Laboratório de Neurociência Experimental e Computacional, Departamento de Engenharia de Biossistemas, Universidade Federal de São João del-Rei, São João del-Rei, Minas Gerais, Brasil; 2 Disciplina de Neurologia Experimental, Escola Paulista de Medicina, Universidade Federal de São Paulo, São Paulo, São Paulo, Brasil; 3 Departamento de Ciência e Tecnologia, Universidade Federal de São Paulo, São José dos Campos, São Paulo, Brasil; 4 Disciplina de Fisiologia, Escola Paulista de Medicina, Universidade Federal de São Paulo, São Paulo, São Paulo, Brasil; Federal University of São Paulo, Brazil

## Abstract

Non-synaptic mechanisms are being considered the common factor of brain damage in status epilepticus and alcohol intoxication. The present work reports the influence of the chronic use of ethanol on epileptic processes sustained by non-synaptic mechanisms. Adult male Wistar rats administered with ethanol (1, 2 e 3 g/kg/d) during 28 days were compared with Control. Non-synaptic epileptiform activities (NEAs) were induced by means of the zero-calcium and high-potassium model using hippocampal slices. The observed involvement of the dentate gyrus (DG) on the neurodegeneration promoted by ethanol motivated the monitoring of the electrophysiological activity in this region. The DG regions were analyzed for the presence of NKCC1, KCC2, GFAP and CD11b immunoreactivity and cell density. The treated groups showed extracellular potential measured at the granular layer with increased DC shift and population spikes (PS), which was remarkable for the group E1. The latencies to the NEAs onset were more prominent also for the treated groups, being correlated with the neuronal loss. In line with these findings were the predispositions of the treated slices for neuronal edema after NEAs induction, suggesting that restrict inter-cell space counteracts the neuronal loss and subsists the hyper-synchronism. The significant increase of the expressions of NKCC1 and CD11b for the treated groups confirms the existence of conditions favorable to the observed edematous necrosis. The data suggest that the ethanol consumption promotes changes on the non-synaptic mechanisms modulating the NEAs. For the lower ethanol dosage the neurophysiological changes were more effective suggesting to be due to the less intense neurodegenertation.

## Introduction

Alcoholism is characterized by addiction to alcohol and persons with this addiction crave alcoholic beverages and develop tolerance to its intoxicating effects [1], [2], [3]. Recent studies indicate alcohol as one of the psychotropic drugs of greater negative impact on public health, personal safety and social structure [4]. In addition, chronic alcoholism can induce alterations in the function and morphology of most if not all brain system and structure [5]. Regarding neuronal degeneration processes, a number of reports have associated effects on memory and learning abilities with special emphasis on hippocampal formation. Therefore, investigations on the role of alcohol in this region are important to understand the signaling pathways and molecular mechanisms involved in these processes [6], [7]. Unfortunately, changes in electrophysiological, morphological and neurochemical brain systems become even more heterogeneous when considering the relationship between alcohol and epilepsy. As we know, alcohol and epilepsy are complexly interrelated and have been linked since Hippocrates [8]. Experimental and clinical studies have been shown that seizures may occur during alcohol intoxication [9], [10] and that people with epilepsy who drink moderate or heavy amounts of alcohol could increase the risk of seizures [8]. Moreover, alcohol is a risk factor for ischemic cerebral infarction [11] and increases the chances of head trauma [12], both of which are known factors in inducing epilepsy.

Currently, it is very important to note that most research in these specific areas are based on strong body of studies that highlights synaptic changes promoted by ethanol showing evidence of altered neuronal communication mediated by acetylcholine, dopamine, serotonin, glutamate, GABA and other neurotransmitters, by direct and indirect action [13], [14]. In this line of reasoning, despite the important findings about the ethanol effects on synaptic functioning, the non-synaptic actions are also capturing important and interesting attention. The solvent-like actions of ethanol fluidizing the neuronal plasma membrane are being deployed as an interfering factor on the ionic transport mechanisms [15]. Ethanol has also been shown to cause damage to the brain. These effects are proposed to be responsible for glial swelling, brain hydration and neuro-inflammation [16]. In addition, research on the effects of ethanol on the brain has shown a link between non-synaptic mechanisms and epileptiform activity [17], [18].

Epileptiform activity may be erupted along intoxication and alcohol abstinence and may also involve hyper-excitable processes, identified experimentally by enhanced evocate responses and reduced threshold for spontaneous and induced seizure [19]. Increased ionic fluxes are being associated with altered membrane permeability and may be the cause of the increased cellular activity [20], [21]. When inspecting these aspects of the alcohol actions, it is possible to draw similarities between alcohol neurotoxicity and mechanisms involved in the status epilepticus models [19], [22], [23].

Non-synaptic mechanisms are being considered the common factor of brain damage in status epilepticus and alcohol intoxication [17], [24], [25]. In such cases, there is involvement of processes triggered by the collapse of the neuronal cytoskeleton, oxidative stress and lipid peroxidation of cell membranes, producing arachidonic acid [26] and subsequent conversion into prostaglandins. The release of prostaglandins triggers inflammatory processes and, according to Morales-Aza and colleagues [27], during inflammation may occur changes in the chloride co-transporters expression with consequent induced excitability increase. According to computational simulations [28], the latency of the non-synaptic epileptiform activities (NEAs) is modulated by the chloride intracellular accumulation, which is sustained by the dynamic equilibrium established by potassium-chloride cotransporter (KCC2) and sodium-potassium-chloride cotransporter (NKCC1). When the chloride accumulation is enough to promote the Nernst potential positivity in respect to the membrane potential, the excitation increases and sustains spontaneous epileptiform events [29].

Based on these facts and considering that epilepsy and alcoholism are chronic diseases highly prevalent in the world population, the present work reports the influence of the chronic use of ethanol on epileptic processes sustained by non-synaptic mechanisms.

## Materials and Methods

### Animals

All experiments were performed according to protocols approved (3/2011) by the institutional animal care and use committee (CEUA of the Federal University of São João del-Rei). Water and lab chow were provided ad libitum, and the animals were maintained in a temperature (20–22°C), light (12 h), and humidity (50–55%) controlled room.

Six weeks old male Wistar rats were randomly divided in four groups with six animals: control (C), ethanol 1 (E1), ethanol 2 (E2) and ethanol 3 (E3). Groups E1, E2 and E3 were treated daily with a solution with 30% v/v of ethanol, administered by oral gavage, according to the volumes of dosing solution, respectively, 1,0 g/kg, 2,0 g/kg and 3,0 g/kg. The animals of the group C received the volume of solution, however, with saline solution replacing the ethanol. After 28 days of ethanol administration, the animals were euthanized for the brain isolation, hippocampus slicing and posterior electrophysiological recordings and histological evaluation.

### Slice Preparation

Hippocampal transverse slices were prepared at 4°C using a tissue chopper. Slices were stored in an oxygenated holding chamber for ≤1 h before electrophysiological recording. All recordings were acquired in an interface-chamber continuously perfused with artificial cerebrospinal fluid (ACSF) at 32°C under a stream of humidified carbogen gas (95% O_2/_5% CO_2_). Composition of the ACSF was (in mM): NaCl 127, KCl 2, MgSO_4_ 1.5, KH_2_PO_4_ 1.1, NaHCO_3_ 26, CaCl_2_ 2 and glucose 10. All solutions were constantly bubbled with carbogen, keeping the pH at 7.4.

### Non-synaptic Epileptiform Activity Induction

Recording electrodes were made of microfilament capillary thin-walled glass (0.9 mm ID, 1.2 mm OD – Clark Electromedical Instruments, GC150F-10), pulled with a DMZ-Universal Puller (Zeitz-Instruments, Germany). Electrodes were filled with 2 M NaCl (5–10 M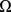) and connected to a headstage (model AI 402×50, ultralow noise amplifier – Axon Instruments, USA). The headstage was connected to a biological amplifier (model Cyberamp 380– Axon Instruments, USA) adjusted with a total gain of 500 and lowpass filtered with cutoff frequency of 3 kHz. The amplified signal was digitized (sampling frequency of 10 kHz) using a system developed in Labview Platform (National Instruments, USA). Recording electrodes were placed in the granular layer (GL) of the DG, at the center of the maximum site of activity identified by means of intrinsic optical signal imaging, according to Almeida et al [30]. Changing bath solution to ACSF containing zero-added calcium and high potassium induced NEAs. The potassium was raised to 8 mM to induce NEAs in the DG. The NEAs usually takes more than an hour to appear, but once it appears, the interval between field bursts and the burst duration remain stable for many hours [31], [32], [33]. Data was analyzed in terms of the following parameters: DC potential shift (DC), event duration (ED), population spike (PS), time between events (TBE) and latency of onset (LO).

The parameter analysis of the extracellular potential recorded was carried out using 15 samples of each experiment. The events selected correspond to approximately 20 minutes after the onset of the NEAs. The parameters extracted from the extracellular potential were: latency (LT), DC shift (DC); maximum amplitude of population spikes (PS); event duration (ED); interval between events (TBE). LT corresponded to the time period between the beginning of the perfusion with zero-calcium solution and the onset of NEAs. The Digital Fourier Transform was used to quantify the DC-shift. Once in the frequency domain, the signal corresponding to the event to be analyzed was recalculated considering only the components inferior to 10 Hz. This process allows reconstructing the event without population spikes. From this signal, the DC could be determined avoiding a subjective criterion for determining the parameter. On the other side, with the signal reconstruction considering only the components superior to 10 Hz, the maximum PS was also measured. The instant of time where the negative deflection reaches 20% of the DC was considered the initial time and the instant of time where the negative deflection returns to 20% of the DC was considered the final time. ED was calculated by subtracting the final time from the initial time of an event. TBE was calculated subtracting the initial time of an event and the final time from the preceding event. These parameters were based on the study conducted by Hoper and colleagues [34].

### Histology

After electrophysiological recordings, slices were post-fixed overnight in 4% paraformaldehyde and embedded in agar gel for posterior resection to slices of 10 µm, on a vibroslicer (VT-100– Leica). After mounting slices onto gelatin-coated slides, they were stained according to Nissl method [35] to assess the cellular boundaries and to estimate the number of cells in DG. After resection, each 400 µm slice yielded, approximately, six 10 µm slices.

### Quantitative Cell Counting

A computational system for image capture and cell quantification was developed in our laboratory. This system includes one microscope coupled with a CCD camera and linked to a computer. This computational system helps the nucleolus identification by means of local magnification around the cell visualized. It also helps the counting process dividing the granular layer in rectangular windows, being each window divided in quadrants. Thus, each cell identified with nucleolus can be marked for counting. Only the cells not touching the right lateral and the inferior borders of each quadrant were counted [36], [37]. The quantification process was carried out separately for the infra- and supra-pyramidal blades and within the electrode neighboring radius of 50 µm. The cell density estimate was calculated by
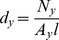
where N_y_ and d_y_ were, respectively, the number of cells and the cellular density on the GL. ‘l’ is the slice thickness (10 µm) and ‘A_y_’ the regional area. Within the region ‘y’, the system allows the operator to draw a polygon that delimits the granular layer. An automatic counting of the pixels within the polygonal allows calculating Ay. For the dark cells quantification the same process was used however counting the number of cells stained dark and with shrunken morphology.

### Immunocytochemical Staining

Additional 6 animals per group, after 28 days of alcohol administration, were euthanized via ketamine-xylazine overdose (100 mg/kg–5 mg/kg, respectfully) followed by transcardial perfusion with 0.1 M phosphate buffered saline (PBS; pH 7.4) and then with 4% paraformaldehyde. Brains were dissected out, post-fixed in 4% paraformaldehyde for 24 h, washed, and stored in PBS at 4°C until sectioning. Brains were cut at 40 µm on a vibrating microtome (Leica Microsystems, Germany).

Vibratome tissue sections (40 µm) from the hippocampus were pretreated with 1% hydrogen peroxide to inactivate endogenous peroxidase activity. The sections were preincubated for 4 hours at 22°C in blocking solution (PBS containing 5% BSA plus normal bovine serum and 0.1% Triton X-100). After, the sections were divided in four groups incubated overnight at 4°C in an affinity - purified anti-KCC2 rabbit polyclonal antibody (1∶200; Abcam), T4 anti-NKCC1 mouse monoclonal antibody (1∶100; Departamental Studies Hybridoma Banck Iowa City, IA), anti-GFAP (Glial Fibrillary Acidic Protein – Astrocytic marker) rabbit monoclonal antibody (1∶1000; Abcam) and anti-CD11b (Integrin αM [CD11b] - Clone OX-42– Microglial activation marker) mouse polyclonal antibody (1∶500; Abcam). Tissue sections submitted to anti-KCC2 and anti-GFAP antibodies were washed and incubated with biotinylated goat anti-rabbit secondary antibody (1∶200, Abcam), and sections submitted to anti-NKCC1 and anti-CD11b antibodies were washed and incubated with biotinylated goat anti-mouse secondary antibody (1∶200, Abcam), both for 2 h at room temperature. The detection of antibodies was performed by the indirect immunoperoxidase method, using kit ABC Elite PK 6100 (1∶200; Vector, EUA). Color reaction was developed with 0,05%, diaminobenzidine tetrahydrochloride (DAB; Sigma Aldrich) and 0,1% H_2_O_2_ in 50 mM Tris-HCl buffer (pH 7.4). Sections were mounted onto glass slides, dehydrated, cleared with xylene and covered with coverslips, using Entellan (Merck).

The quantitative analysis was based on the measurement of the KCC2, NKCC1, GFAP and CD11b immunoreactivity intensity in terms of the percentage optical absorption (POA). The data of the intensity of immuno-histochemical staining were performed by means of a light microscope (AXIO, Carl Zeiss, Germany).

### Optical Densitometry

The immunoreactivity of NKCC1, KCC2, GFAP and CD11b were quantified by optical densitometric analysis. The transversal sections of the hippocampus (n = 6) were cut at 40 µm using a vibroslicer. The slices were photographed with a video camera connected to a microscope. The images were then processed in RGB then compressed to gray scale (RGB mean band); afterwards the corresponding histograms were obtained. In order to increase the contrast, histogram equalization technique [38] was incorporated into the process. The resulting images provided better visualization of the stains, resulting in more reliable segmentations. A gray scale range was defined as a threshold to consider the tissue stained area (between 0 and 65 in gray scale). The significant pixels were then converted into binary matrix (black and white) and quantified by the black pixels sum per area. The quantification was developed by MATLAB, under the same resolution images and same optical enlargement. The data was plotted as percentage corresponding to the equivalent stain of the image section. The results of the optical densities were analyzed with one-way ANOVA with Dunnett’s test and values of p<0.05 were considered statistically significant. The data were plotted considering control group = 100%.

## Results

### Alcohol Long-term Intoxication Modulates Non-synaptic Activity

**Figure 1:**
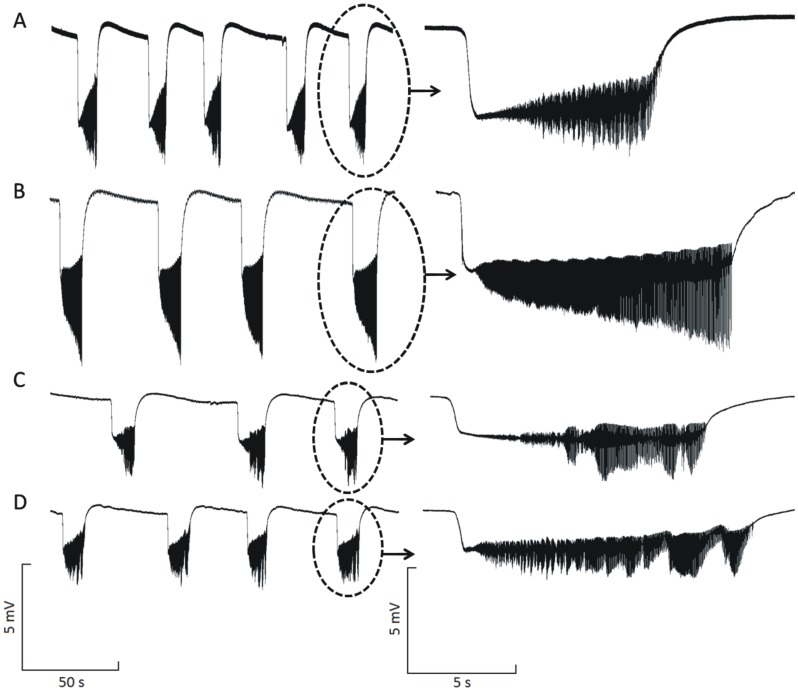
Typical non-synaptic epileptiform activities induced in hippocampus slices and recorded in the granular layer of the dentate gyrus according to the groups. (A) control, (B) E1, (C) E2 and (D) E3.

**Figure 2:**
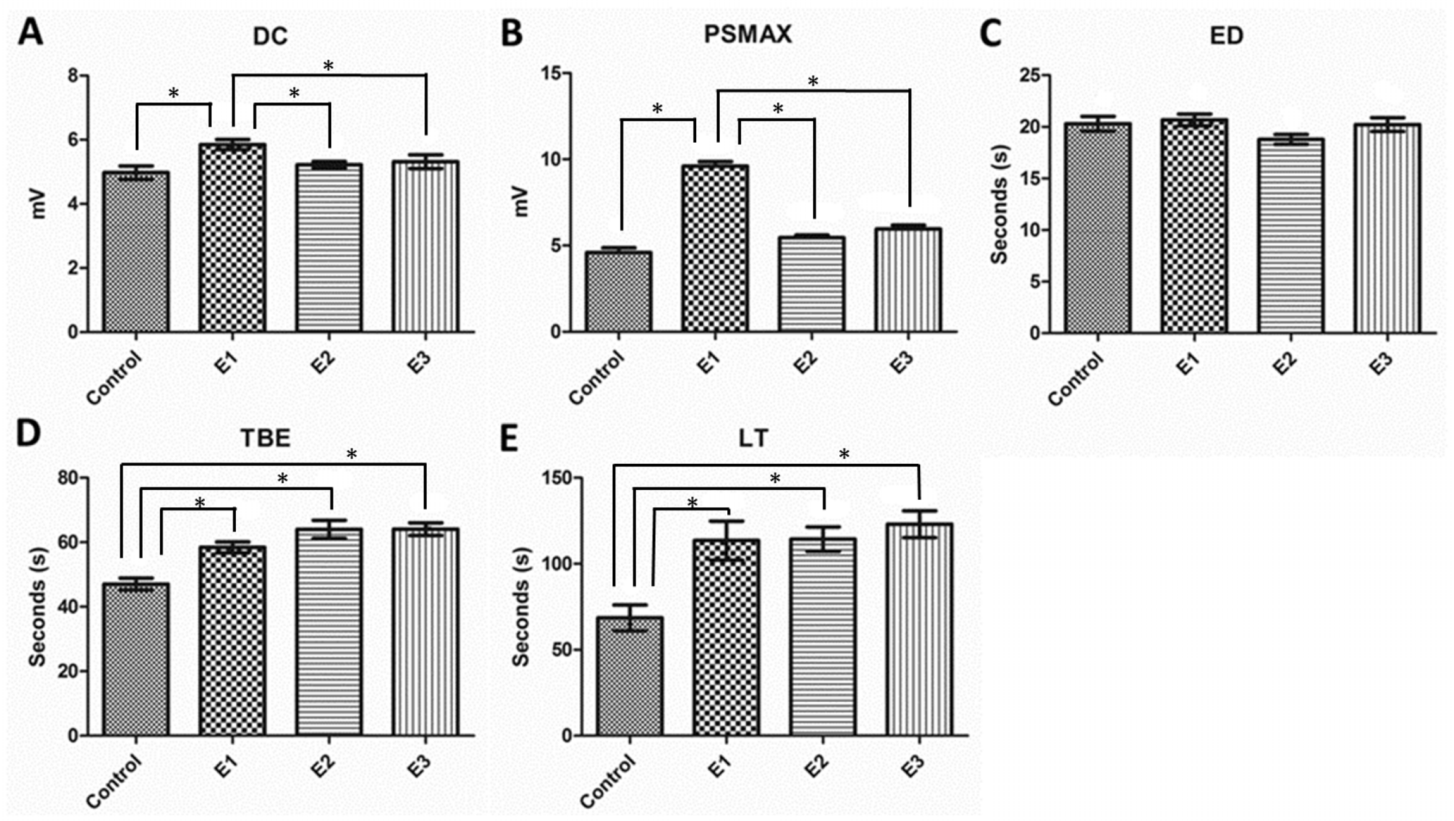
Parameter analysis of the extracellular potential recorded for non-synaptic epileptiform activities. Statistical comparisons were done with one-way ANOVA with Dunnett’s test. A: Average amplitude of DC shift during burst activity. E1 group showed the highest value, while control, E2 and E3 did not differ statistically. B: Maximum population spike (PS). E1 showed the highest value, while control, E2 and E3 did not differ statistically. C: Events durations (ED). No difference was found between all groups. D: Time between events (TBE). Control exhibited the lowest value, while E1, E2 and E3 did not differ statistically. E: Latency (LT) to the onset of the non-synaptic activities. Control exhibited the lowest value, while E1, E2 and E3 did not differ statistically. Data are presented as mean ± SEM. Error bars indicate SEM. ^*^ *p*<0.05.

Twenty minutes after the onset of the NEAs, induced with low calcium and high potassium, fifteen epileptiform events (∼ 6 min of record) were quantified for each experiment (Table 1). The basic morphological differences observed are illustrated in Figure 1 (n = 6, for each group). The highest DC shift and PS were encountered for the E1 group, which was significantly different from the other groups (Figure 2A and 2B). Comparing the groups in terms of the duration of the ictal periods, defined by the parameter ED, no significant differences were found (Figure 2C). On the other hand, the interictal periods, defined by the parameter TBE, showed significant difference when comparing the treated groups with the Control. Between the treated groups, the difference was significant only when comparing E1 and E2 (Figure 2D). The time demanded to induce the non-synaptic activity after Ca^2+^ deletion and K^+^ increase, defined by the parameter LT, was increased for all groups (Figure 2E).

**Table 1:** Parameter quantification of the non-synaptic epileptiform activity.

Groups	DC	PS	ED	TBE	LT
Control	4,974±0,209	4,591±0,276	20,29±0,726	47,06±1,846	68,50±7,52
E1	5,853±0,157	9,601±0,264	20,67±0,562	58,42±1,692	113,5±11,28
E2	5,224±0,098	5,457±0,151	18,78±0,478	64,02±2,784	114,3±7,12
E3	5,315±0,213	5,956±0,222	20,22±0,672	64,09±1,978	123,0±7,78

Data are expressed as mean ± S.E.M.

### Granular Cell Layer Morphology Changes with the Chronic Ethanol Consumption

Since morphological changes have been associated with the prolonged use of alcohol in high doses [39], [40], histological sections stained by Nissl method were analyzed from hippocampal slices submitted and not submitted to NEAs induction.

**Figure 3:**
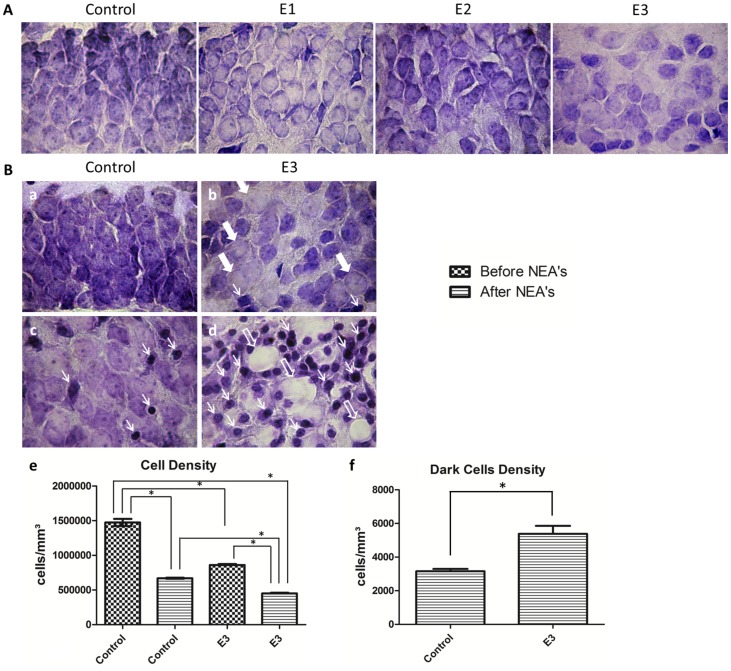
Nissl stain analysis. A: After Nissl staining, the typical morphology of the granule layer of DG for each group investigated (control, E1, E2 and E3) are shown. B: Effect of the non-synaptic activity induction on the cell morphology, performed for the group E3 in comparison with control. Before seizure induction, the normal morphology of the granule layer (a) is replaced by the presence of whitish zones, somas with swelling (thick arrows) and dark cells (thin arrows) (b). After seizure induction, the slices of the control group showed edematous cell bodies and a few dark cells (thin arrows) (c). However, the slices from the group E3 appeared with intense edematous necrosis, as shown in (d). Several cell bodies with intense swelling (thick arrows) and dark cells (thin arrows) can be seen.

Sections from animals of the groups E1, E2 and E3, which slices were not submitted to epileptiform activity induction, showed a visible cell loss and morphological alteration of the DG granular layer (Figure 3A). The DG cytoarchitecture of the E1 group appeared more preserved than the other experimental groups. However, the granular cells in dentate gyrus showed altered morphology with increased cell size. In E2 and E3, whitish regions were observed characterizing cell dispersion along the dentate gyrus. The sections of the E3 group showed the higher dispersion when compared with cell groups E2, E1 and control.

The cell density of the granular layer was quantified for all groups investigated. Groups E1 (1101000±33920 cell/mm^3^), E2 (1052000±81630 cell/mm^3^) and E3 (860500±15150 cell/mm^3^) showed significant reduction in the number of cells per slice, compared with the control group (1474000±51800 cell/mm^3^). In the groups E2 and E3 this reduction was even greater, when compare with control and E1 groups.

Furthermore, the presumed predisposition of the alcohol treated group to aggravate histological changes under seizure induction, justified to investigate the group E3 in comparison with Control after NEAs induction. Significant histological changes were observed in slice submitted to induction of NEAs, characterized by cell density reduction, whitish zones and significant increase of the density of dark cells (Figure 3B). The typical cell morphology observed showed pyknotic nucleus and condensed chromatin resembling irregular dispersed agglomerates.

### Immuno-histochemical Analysis Associate Alcohol Chronic Consumption with Changes in Non-synaptic Mechanisms

**Figure 4:**
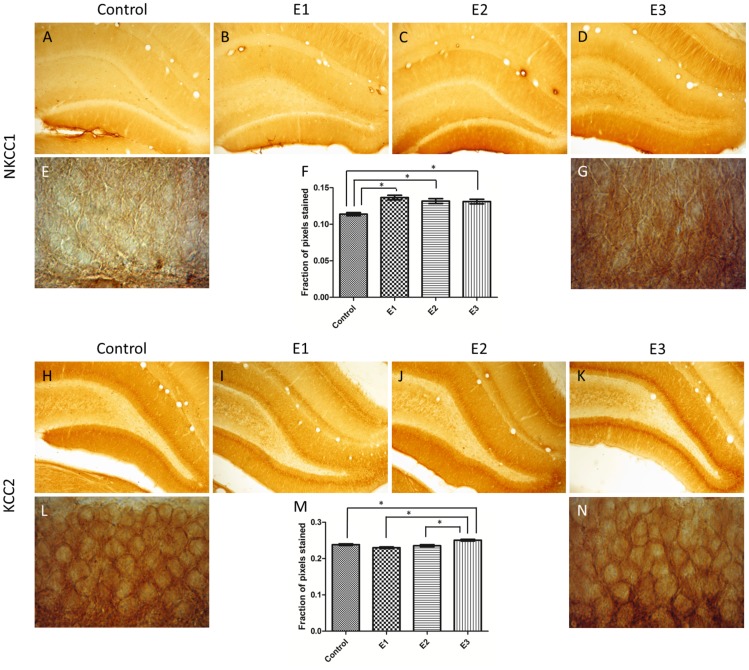
DG immuno-stained for NKCC1 and KCC2. Typical images for NKCC1 (Magnification: 400x). All alcohol treated groups expressed more intense staining for NKCC1 than Control (A). For KCC2 the immuno-reactivity was more intense for the group E3 (K) (Magnification: 1000x). The transition between the granule and molecular layers exhibited the most intense staining for both NKCC1, comparing E3 (G) with Control (E), and KCC2, comparing E3 (N) with Control (L). Statistical comparisons were done with one-way ANOVA with Dunnett’s test. Data are presented as mean ± SEM. Error bars indicate SEM. *p<0.05.

**Figure 5:**
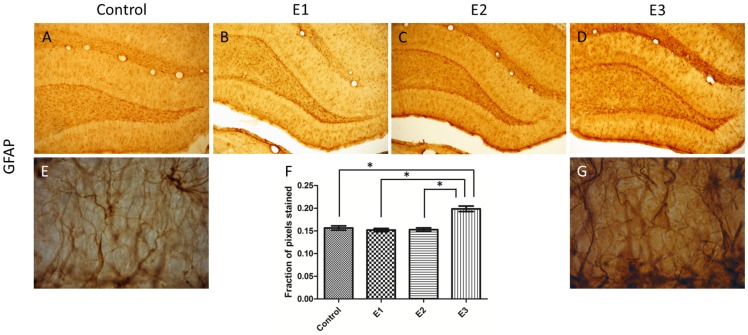
DG immuno-stained for GFAP. (A) Control section, (B) E1 section, (C) E2 section, (D) E3 section (Magnification 100x). The astrocyte reactivity typical of E3 (G) is absent in the control (E) (Magnification 1000x). (F) Optical densitometry analysis of GFAP immuno-staining showed E3 with significant increase in comparison with the other groups. Statistical comparisons were done with one-way ANOVA with Dunnett’s test. Data are presented as mean ± SEM. Error bars indicate SEM. *p<0.05.

**Figure 6:**
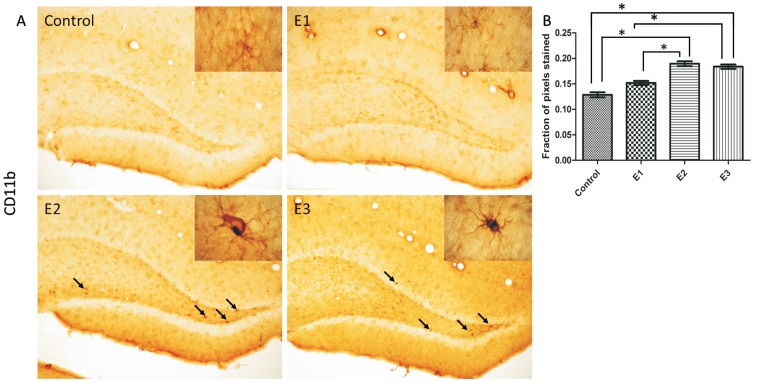
DG immune-stained for CD11b. (A) Control, E1, E2 e E3 (Magnification 100x). Insets show typical microglial morphology of each group (Magnification 1000x). Arrows head indicate microglial activation also shown in the insets. (B) Optical densitometry analysis of the immuno-reactivity to CD11b. E2 and E3 groups exhibited increased immuno-staining in comparison with Control and E1. Statistical comparisons were done with one-way ANOVA with Dunnett’s test. Data are presented as mean ± SEM. Error bars indicate SEM. *p<0.05.

Immuno-histochemical staining was performed in the DG for NKCC1, KCC2, GFAP and CD11b. The quantification by optical density analysis was summarized, respectively, in Figures 4, 5 and 6. The treated groups, E1, E2 and E3 exhibited NKCC1 staining more intense than Control (Figure 4, A–G). The staining for KCC2 was more intense for the group E3, when compared to E1, E2 and Control (Figure 4, H–N). In all sections stained with anti-NKCC1 and anti-KCC2 an intense immuno-reactive pattern was concentrated in the transition between the granule layer and molecular layer.

In samples stained with anti-GFAP, GFAP-positive astrocytes were visible in the hilus, exhibiting small, round and thin cytoplasmic processes. The alcohol treated groups exhibit astrocyte morphological changes that seem to be dependent on the alcohol concentration of the treatment (Figure 5). A qualitative inspection suggested astrocyte activation with hypertrophy of the cell body, aberrant cytoplasmic processes and increasing immuno-staining intensity dependent on the alcohol concentration. However, the statistical analysis showed that the differences between groups are significant only for the group E3 (Figure 5). These characteristics of the astrocyte activation were visualized preferentially in hillus with cytoplasmic projections invading intensely the granule layer. It is also expressed, however less intense, in the transition between the granular and molecular layers.

The microglia activity was examined by means of immuno-staining with CD11b. The expressions of CD11b in the alcohol treated groups were compared between the groups exhibiting increased expression of E2 and E3 in comparison with E1 and Control (Figure 6). The groups Control and E1 showed quiescent microglias with soma small and compact with thin ramified processes, on the other hand, the microglias of the groups E2 and E3 exhibited intense immune-reactivity characterized by active phenotype with remarkable cellular hypertrophy and retraction of the cytoplasmic processes.

## Discussion

For the first time, to our knowledge, it was shown the chronic alcoholization and its effects on the NEAs induced in hippocampus slice. This work has two primary findings. First, hippocampal slices of rat submitted to chronic alcoholization exhibited changes in NEAs induced by high K^+^ and zero-Ca^2+^ perfusion. Second, granule cell layer of the dentate gyrus showed severe edema and necrotic aspect after induction of NEAs in the slices of the alcoholized animals.

These changes were associated with changes in the expression of cotransporters (NKCC and KCC), astrocytes, microglia and cellular density. Relation between both findings may be inferred based on biophysical mechanisms involved in the generation of the NEAs and in the effects of the alcohol on the non-synaptic processes according to the morphological analysis.

The NEAs induction promoted significant increase of the parameters DC and PS of the E1 alcoholized group in respect to Control, thus, demonstrating the functional changes of the nonsynaptic mechanisms being affected by the alcohol intoxication. These changes were less intense for the groups E2 and E3 for which higher dose of alcohol were used. Cohen and colleagues [41] also described this biphasic effect of ethanol across the ethanol concentrations. The present result reports E2 and E3 groups with more intense cellular density reduction then E1. Despite the fact that ethanol has many different pharmacological actions [42] the observed biphasic pattern of NEAs potentiation seems also to be dependent on the neuronal damage. The decreased intensity of the NEAs of the groups E2 and E3 when compared to E1 may also be attributed to the more pronounced neuronal loss in the DG layer. In line with these findings is the increased latency to seizure induction, expressed by the parameter LT, suggesting that the neuronal damage delayed the seizure eruption. According to computational simulations of NEAs [28] if the cell density reduction is associated with extracellular space increase, therefore the DC level reduction, observed when comparing E2 and E3 with E1, could be explained by an associated decrease of the ionic fluctuation at the extracellular space, which in turn reduces the DC shift. Taking into account that an increase in extracellular space is followed by an extracellular resistance decrease, the PS reduction, when comparing E1 with E2 and E3, may also be explained. According to Fox and colleagues [43], the increased proximity of neurons increases the extracellular resistance within the granule layer, which increases the efficacy of field effects between adjacent granule cells. Therefore, it is plausible to suppose that the PS observed for E1, greater than that observed for E2 and E3, is due to changes in spacing between cells. However, the Control group showed the highest cell density with the lowest PS, which seems to be a contradiction. But, the investigation of slices submitted to NEAs induction (Figure 3) showed intense edematous process, meaning that the cell swelling, promoted along the seizure induction, reduces the cellular spacing, presumably even more than the Control. This observation offers a reliable explanation about the higher PS found in the alcoholized groups in comparison with the control.

The extracellular changes inferred by the cell density changes and the cell swelling due to the edema promoted by the induced seizure affect directly the extracellular ionic fluctuations, supporting faster changes for the same ionic fluxes. Inversely, increase in extracellular space slows down the effects on the extracellular ionic concentrations. Changes in the intra and extracellular ionic concentrations modulate the dynamic equilibrium of the transmembrane ionic gradients regulated by the coupled functioning of the Na/K pump and cotransporters. The simulation of these mechanisms shows that when the extracellular K^+^ increases, a Cl^−^ influx takes place and may be the main responsible for the increasing excitation, able to sustain spontaneous epileptiform activities [28]. Despite the complexity involved, the observed changes in the expression of NKCC1 and KCC2 allow drawing a plausible explanation for the changes observed in the parameters LT and TBE.

The evident changes in the granular layer, with the soma swelling of the granular neurons, indicate extracellular reduction for the alcohol treated groups and allow proposing an explanation for the increased latency to the onset of the NEAs, indicated by the significant increase of LT for E1, E2 and E3 in respect to the Control group. The reduced extracellular volume subserves a more effective controlling of the extracellular K^+^ level by the complex interplay of the Na^+^/K^+^ pump and the cotransporters. Cl^−^ intrusion occurs coupled to the increased extracellular K^+^. The increased intracellular volume demands longer time to increase the intracellular Cl^−^. Therefore, longer time will be required to enhance the excitability, resulting increased LT.

The occurrence of cell degeneration in the granular layer of the GD, shown by the histological analysis, makes evident the inflammatory process due to alcohol toxicity, indicated by the increased expression of microglia and also the reactive astrocytosis, as shows the immuno-staining with CD11b and GFAP, respectively. These increases in microglia and astrocyties are being correlated with the occurrency of glioses, with astrocytic activation pattern indicating formation of astroglial scar [44] and phagocytic activation [45]. These findings support the hypothesis proposed by Valles and colleagues [46] associating the alcohol consumption to the enhanced inflammatory effect and the enlarged glial proliferation. Recent studies also support the ethanol abuse associated with neuroinflammatory pathway activation [24].

The changes in the interictal period duration, quantified by the parameter TBE, had a non-monotonic behavior in dependence on the ethanol dosage. The interictal period is highly dependent on the ionic equilibrium established between the intra and extracellular spaces following the complex dynamic of the coupled action of the Na/K pump and the NKCC1 and KCC2 cotransporters. Along of the interictal period, the intrusion of Cl^−^ is the main modulator of the excitation and is mainly guided by the interplay between NKCC1 and KCC2 [29], [28]. The alcohol treated groups had significant increase of the TBE parameter in respect to the Control group. The inflammatory processes are known to promote changes in the expression of the chloride cotransporters [27], [47]. Inflammatory process associated with kindling and spontaneous seizures may lead to increased NKCC1 expression [48]. In the alcohol treated groups similar situation where observed. The NKCC1 expression changed significantly comparing treated groups with Control. Since Cl^−^ is carried into the cells by the NKCC1, the excitability of the treated groups would be increased with NKCC1 increased expression. The facilitated intrusion of Cl^−^ along the interictal period enhances the transition to the ictal state, exhibiting diminished duration of this period.

Slices from the alcohol treated groups showed a clear predisposition to edematous necrosis with dark cell degeneration after induction of NEAs. Studies conducted with even shorter period of treatment (one week) also exhibited microglial proliferation and lesions in the central nervous system with cell death provoked by edematous necrosis and also characterized by the presence of dark cells, with clear induction of cellular inflammation [49], [50]. The present data show that the induction of NEAs may lead to necrotic process and may be significantly exacerbated by the alcohol action. According to Collins and colleagues [17], the ethanol use promotes hydric accumulation in neuronal cells, being responsible for irreversible neurodegeneration in limbic cortical regions with lesions followed or preceded by electrolytes accumulation and brain edema. The morphology of the necrotic cells seems to be dependent on the insult. The dark cell degeneration is a type of necrosis associated with neuronal excito-toxicity after *status epilepticus* [51] and the action of AMPA receptor and kainate [52], [53], [54]. Similar to edematous necrosis, the dark cell degeneration is accompanied by pyknotic nuclei with chromatin irregularly fragmented. The basic difference between those types of necrosis is the cytoplasmic changes in dependence on each type of necrosis. During the edematous necrosis, the cells exhibited soma with intense swelling. On the other hand, the dark cell degeneration is associated with cell body shrink and therefore with basophilic aspects characterized by the dark sites. The presented results showed that slices submitted to NEAs induction also showed both histologic changes, suggesting that both types of necrotic processes may occur, confirming findings from Lovinger [55] and Fadda and Rossetti [5].

In line with the present results, Collins and colleagues [17] showed that the swelling processes and the edematous degeneration may be effectively diminished by means of the blockage of the cotransporters NKCC1 and KCC2 with the furosemide. Therefore, the non-synaptic mechanisms, involved with the inflammatory processes and the cell swelling, consequently with the subsequent cellular degeneration, seems to assume the most relevant whole at the initial effects of the alcohol abuse, possibly preparing the scenario for enhancing the excitatory synaptic circuitry. In fact, the excitatory synaptic mechanisms were described occurring later [56], [57].

The evidences of the present report suggest that the changes of the non-synaptic mechanisms induced the group treated with the lowest dosage seems to create suitable conditions to enhance NEAs. Additionally, the changes provided by the alcohol abuse are enough to predispose the brain to develop neurodegeneration once triggered by an eventual epileptic seizure. The NEAs involved with the seizure may reinforce the changes of the non-synaptic mechanisms by means of edematous necrosis and dark cells degeneration. These changes may lead to a scenario with ionic homeostasis favorable to enhance excitation, predisposing the brain to subsequent seizures. The presented evidences motivate the investigation of new strategies to counteract the non-synaptic changes induced by the alcohol intoxication aiming neurodegeneration prevention. Additionally, the findings suggest the increased risk of neurodegeneration when alcohol depending persons are affected by epileptic seizures.
